# Application of Microfluidic Chip Technology in Food Safety Sensing

**DOI:** 10.3390/s20061792

**Published:** 2020-03-24

**Authors:** Hongwei Gao, Chunlei Yan, Wei Wu, Juan Li

**Affiliations:** 1 College of Mechanical and Electrical Engineering, Qingdao Agricultural University, Qingdao 266109, Shandong, China; gaohongwei@qau.edu.cn; 2 College of Food Science and Engineering, Qingdao Agricultural University, Qingdao 266109, Shandong, China; 201901003@qau.edu.cn (C.Y.); wuwei88@qdu.edu.cn (W.W.)

**Keywords:** food pollution detection, microfluidic chip, rapid sensing, multiple detection

## Abstract

Food safety analysis is an important procedure to control food contamination and supervision. It is urgently needed to construct effective methods for on-site, fast, accurate and popular food safety sensing. Among them, microfluidic chip technology exhibits distinguish advantages in detection, including less sample consumption, fast detection, simple operation, multi-functional integration, small size, multiplex detection and portability. In this review, we introduce the classification, material, processing and application of the microfluidic chip in food safety sensing, in order to provide a good guide for food safety monitoring.

## 1. Introduction

In recent years, major food safety incidents have occurred frequently, people are very concerned about the excessive content of metals and additives, pesticide residues and microbial contamination in food, and food safety has become a global topic [1,2,3]. Solving food safety problems requires monitoring the entire process of food production, processing, and distribution from farm to table. Food safety analysis and testing is an important means to control food contamination and supervision [4,5,6]. The traditional detection technology is generally based on instrumental analysis, which is accurate and reliable, but it has some limitations, such as expensive instruments, long cycle, large material consumption, complicated operation and low sensitivity, so it is difficult to meet the needs of the on-site, real-time, rapid and portable detection of food [7,8,9,10]. Therefore, there is an urgent need to meet the needs of on-site, fast, accurate and popular food safety analysis and detection technology.

Microfluidic chip technology integrates the process of sample pretreatment, separation and detection involved in molecular biology, chemical analysis, medicine and other fields into a chip of a few square centimeters, thus realizing the miniaturization, automation, integration and portability of sample pretreatment and follow-up analysis. It involves the intersection of biology, chemistry, medicine, electronics, materials, machinery and other disciplines. With the advantages of less sample consumption, fast detection, simple operation, multi-functional integration, small size and portability, it has been applied in many fields, such as cell biology, genetic analysis, chemical composition analysis and food safety sensing [11,12,13,14]. In 2003, Forbes magazine rated this technology as “one of the 15 most important inventions affecting the future of mankind”. 

The characteristics of microfluidic chip technology are as follows: First, a series of special effects, such as laminar flow, surface tension and capillary effect, rapid heat conduction and the diffusion of microfluidics, which are conducive to accurate fluid control and rapid reaction. Second, the complexity of the structure. The micro processing technology has the ability to process a small-scale, high-density micro structure, which is convenient to realize the flexible combination and scale integration of various operation units. Therefore, the experimental processes of sample pretreatment, separation and analysis, and detection, can be integrated and parallelized on the same chip, so as to achieve the goals of miniaturization, automation, low consumption and high efficiency [15,16]. Microfluidic chip technology is a hot field in the development of a micro total analysis system, and has become one of the most advanced technologies in the world [17,18,19]. However, the high cost of traditional microfluidic chips limits its large-scale application. In 2006, Whitesides et al. [20] first proposed the concept of the “microfluidic paper analysis device”, where the paper-based microfluidic chip has the advantages of low cost, simple preparation, good sensitivity, convenient use and carrying, small volume of the required sample, fast analysis speed, simultaneous detection of a variety of substances and the like, and has shown strong development vitality and good application prospects in the field of food safety detection [20,21,22]. It is expected to become the cheapest means of analysis and detection. This paper focuses on a series of important advances made by microfluidic chip technology in the food safety testing of food ingredients, pesticide residues, pathogenic bacteria, heavy metals and food additives, and prospects for its application in food safety analysis.

## 2. Research on Microfluidic Chip Technology

The research of microfluidic chip technology involves chip classification, processing, sealing, microfluidic driving, signal collection, analysis and detection. The microfluidic chips can be divided into the platinum resistance chip, pressure sensor chip, electrochemical sensor chip, nanoreactor chip and the microflow fuel chip according to the different reaction principles of microfluidic chips. 

The fabrication of the microfluidic chip includes chip processing, sealing and other links, mainly relying on the MicroElectro-Mechanical System (MEMS) processing technology, with the ability to achieve micro-flow control at the micron level. Because of their good biocompatibility, strong plasticity, strong affinity, low cost and simple fabrication process, the Macromolecular polymer materials polydimethylsiloxane (PDMS) and copolymers of cycloolefin (COC) are widely used in the fabrication of biochemical analysis devices [23]. The processing technology of the microfluidic channel includes soft lithography and etching technology, the hot pressing method, molding method, injection molding method, integrated lithography, electroforming and plastic casting (LIGA) method, the laser cauterization method and other traditional methods, as well as new methods, such as 3D printing [24]. The sealing of microfluidic channels can adopt plasma surface treatment or immediate bonding after deep ultraviolet irradiation, ultrasonic welding, laser welding, film sticking and so on, mainly considering aging and pipeline blockage.

## 3. Materials of the Chip

Silicon and glass are the earliest substrate materials used in microfluidic chips, mainly because they can directly use processing methods in the field of MEMS and microelectronics. Silicon and glass materials are expensive and difficult to process, which have been replaced by a variety of low-cost polymer materials, such as elastomer materials [25], thermoplastic polymers [26], thermosetting polymers [27], paper materials [28], biomaterials [29], etc. In this paper, the materials used for low cost microfluidic chip fabrication are divided into polymer materials, paper materials and other materials.

### 3.1. Elastomer Materials

**Figure 1:**
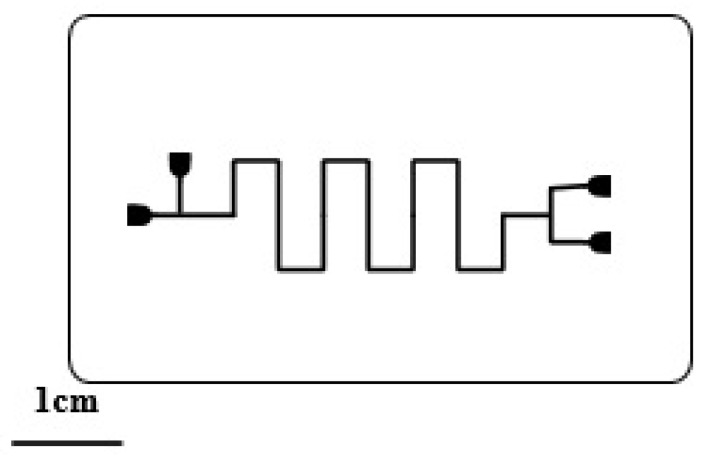
Microdroplet chip based on polydimethylsiloxane (PDMS) material.

The term “elastomer material”, a type of polymer material, that can undergo significant deformation under weak stress, and rapidly return to a state and size close to the original state and size after stress relaxation. PDMS is currently the most widely used elastomer material in the field of microfluidic chips. Its application in microfluidic chips was first proposed by Whitesides et al. [30] in 1998. PDMS has the advantages of low price, optical transparency, good biocompatibility, and a certain degree of permeability [31], and is an ideal material for low-cost microfluidic chips (as shown in Figure 1). PDMS in microfluidic chip processing is often through the molding method in the surface of the formation of microstructure, and the precision of the turnover can even reach nanometer (nm) level [32,33]. However, PDMS also has the channel easy to collapse, a small amount of fluid absorption, and other shortcomings. PDMS processing and bonding methods will be more detailed in the part of low-cost processing in this article.

### 3.2. Thermoplastics

Thermoplastics are the most common and widely used materials in our daily life, and the price is very low. It can be softened and shaped at a certain temperature. Thermoplastic materials that can be used for low-cost, microfluidic chips are many, mainly polymethyl methacrylate (PMMA), polystyrene (PS), COC, polycarbonate (PC), polyterephthalic acid (PET), polyvinyl chloride (PVC), etc. In thermoplastic plastics, PMMA has been widely used in various life sciences [34] and medical research [35], because of its low cost and good thermal processing and optical properties. PS has excellent biocompatibility, and has significant advantages in the field of cell culture [36] as the matrix material of microfluidic chips; COC is a relatively new amorphous copolymer material. Compared with PMMA and other thermoplastic materials, COC chips have excellent transmission performance and better thermal stability in the ultraviolet band, while the water absorption is only 1/10 [37] of PMMA, in most cases (non-extreme temperature cases). It can directly replace expensive glass chips.

### 3.3. Paper Material

**Figure 2:**
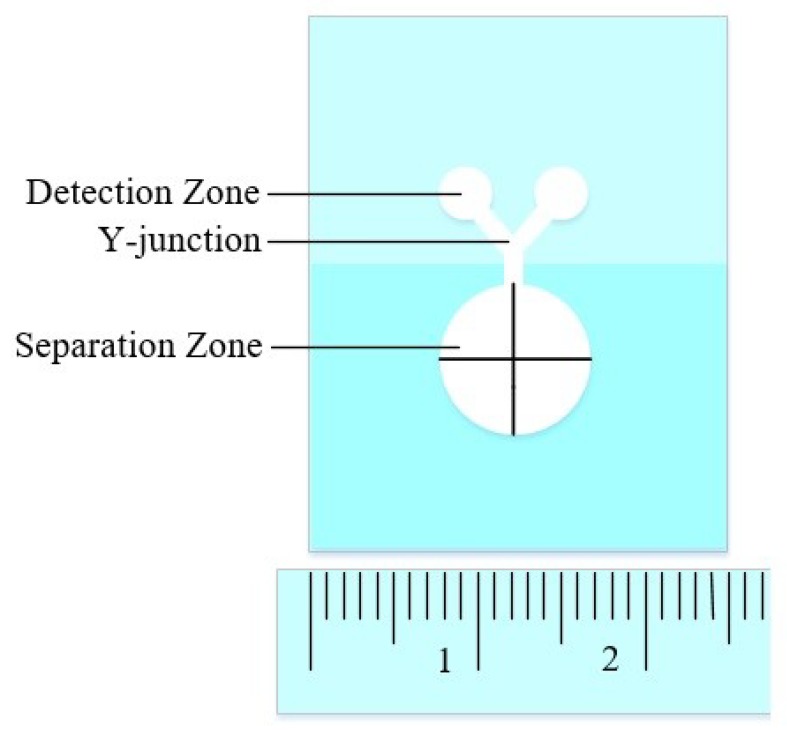
Paper-based microfluidic chip for blood cell separation.

Paper-based microfluidic chip is formed by infiltrating hydrophobic materials into hydrophilic paper fibers through various methods, controlling the fluid flow in hydrophilic paper fibers through the “wall” of hydrophobic materials, thus forming a paper-based microfluidic chip. Common inkjet printers [38], screen printing [39], 3D printers [40], wax printers [41] and even crayons [42] can be used to process low-cost, paper-based microfluidic chips. In the choice of paper, the common ones are Whatman series filter paper [43] or chromatographic paper [44]. Unlike polymer microfluidic chips, which need to close the channel, paper-based microfluidic chips often do not need to close the channel; that is, they are open-channel, because the liquid moves inside the paper fibers. The paper-based microfluidic chip for blood cell separation and serum protein detection shown in Figure 2. 

It defines the channels through which the liquid flows in the paper fibers by means of dipping wax, then separates the blood plasma and blood cells through the pores of the paper fibers, and finally determines the content of serum protein by means of coloration. Paper-based microfluidic chips have been widely used in various medical and life science detection research and applications, due to the low cost of materials and processing, such as saliva acetaldehyde detection [45], heavy metal detection [46], blood sugar detection [47], lactic acid detection [48], etc.

## 4. Processing Method of Chip

### 4.1. Micro-Molding

Due to the wide application of PDMS materials in the field of microfluidic chip processing, micro-molding based on PDMS has become the most common microfluidic chip processing method. Among them, it is common to use SU-8 photoresist as a mold to mold PDMS [49,50]. The SU-8 photoresist is spun-coated on a silicon chip and photoetched, and its thickness can be freely adjusted within a range of more than 10 to 200 microns, according to different types of SU-8 photoreceptors and the control of the spun-coating speed. The PDMS and hardener were mixed in a ratio of 10:1 to remove the air bubbles, then the mixture was slowly poured onto the SU-8 microstructure and heated to harden; the PDMS was carefully removed from the SU-8 mold, and the mold could be reused; the PDMS was bonded with glass substrate after oxygen plasma treatment.

### 4.2. Laser Ablation

The laser ablation here specifically refers to a method of ablating and machining a micro flow channel on the surface of a polymer material by using a carbon dioxide laser with a wavelength of 10.6 μm [24]. 

The laser ablation method for machining the micro flow channel has the advantages of simple, fast, and one-time ablation to complete the processing. This method is widely used, and most polymer materials and glasses can use the method to machine the micro flow channel on the surface. Disadvantages are that: The inner wall of the micro-channel machined on the surface of the polymer material is uneven, and there are a large number of bubbles, which may need to be treated by chemical methods [51]. There are protrusions formed by the casting and re-solidification of molten material on both sides of the flow channel machined on the surface of the polymer material, which is not conducive to subsequent bonding, and the processing accuracy is limited, which is only suitable for applications where the width and depth of the flow channel are greater than 80 μm. The application of the laser ablation method in the field of low-cost microfluidic chips is still focused on the application of a single polymer material, and from the future development direction, there is still a large space for development in the field of microfluidic chip processing based on biodegradable plastics, paper, conductive plastics and other materials.

### 4.3. 2D/3D Printing

**Figure 3:**
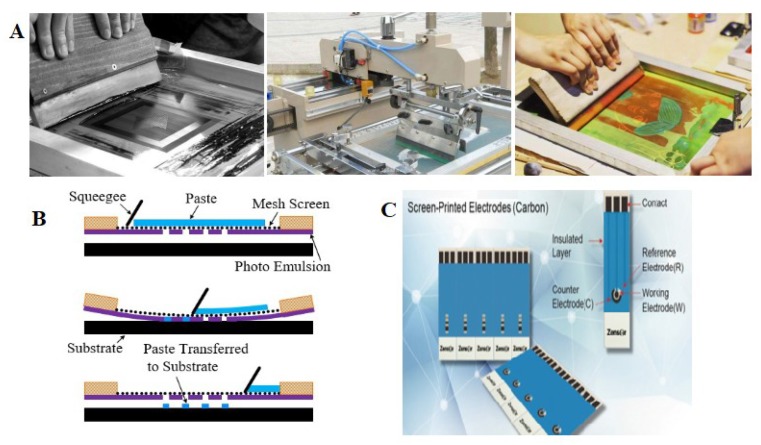
Microfluidic chip based on screen-printing: (**A**) the Screen-printing device; (**B**) the principle of screen-printing; (**C**) the screen-printed electrodes.

2D printing refers to a method for processing a microfluidic chip or a microfluidic chip pour-back mold, which is common in office and experimental occasions, such as a laser printer [52], an inkjet printer [53], a wax printer [54], a screen printing [55], etc. 3D printing refers to a technology [56] for directly printing a microfluidic chip or a pour-back mold by using a 3D printer that has developed rapidly recently. 2D-printed microfluidic chips are usually used in paper-based microfluidic chips, which are surrounded by hydrophilic paper materials by the impregnation of hydrophobic ink to form microchannels, and the pattern accuracy is determined by the printer accuracy or screen mesh, usually between 80 and 400 μm. In addition, microstructures made of PDMS, SU-8 or the like can be directly deposited on a glass or polymer substrate by ink-jet printing or screen printing to form microfluidic chips [56,57], and electrodes can be printed on the surface of the microfluidic chips if conductive ink containing silver nanoparticles is used [58]. The basic processing model of screen printing was shown in Figure 3 [59], and the microchannel and silver electrode based on UV sensitive medium slurry (5018a, DuPont, USA) processed by screen printing method [60]. Moreover, new type of printing method was used on electrodes (Figure 3C).

The main methods of using 3D printing to process microfluidic chips are stereo-lithography [40] and fused deposition modeling (FDM) [61], among which the fused deposition modeling 3D printer can be used to process low-cost 3D microfluidic chips because of its relatively low price. The fused deposition molding technique can be used to directly print PC, PLA (polylactic acid), ABS (acrylonitrile butadience styrene), and other materials to make 3D microfluidic chips [62], and can also be used to print the mold for the PDMS reverse mold [63]. However, the accuracy of commercial fused deposition molding equipment is between 100–500μm, which is still far from the application requirements of most microfluidic chips, and the selection of transparent printing consumables suitable for microfluidic chips is limited, and the processing speed of chips is slow compared with other methods introduced in this paper.

### 4.4. Injection Molding

Injection molding is a widely used processing method in the field of plastic processing. In recent years, with the development of micro-injection molding technology, researchers began to try to use this injection molding method to process microfluidic chips. The common injection materials used in microfluidic chips are PMMA, COC and PDMS [64]. Traditionally, processing microfluidic chips by injection molding requires processing molds first, which is expensive and time-consuming. In order to reduce the cost of microfluidic chip fabrication, Hansen et al. [65] used su-8 photoresist on the surface of nickel as an injection mold, and the mold could be reused for 300 times, which has greatly reduced the cost and time of mold fabrication. The advantage of that invention lies in good repeatability, fast processing speed, that it can process 3D microfluidic chips, and is suitable for processing large-scale microfluidic chips; the disadvantage is poor flexibility, the need to re-open the mold when the chip structure changes, and the mold cost is high.

### 4.5. Low-Cost Microfluidic Chip Bonding Technology

**Figure 4:**
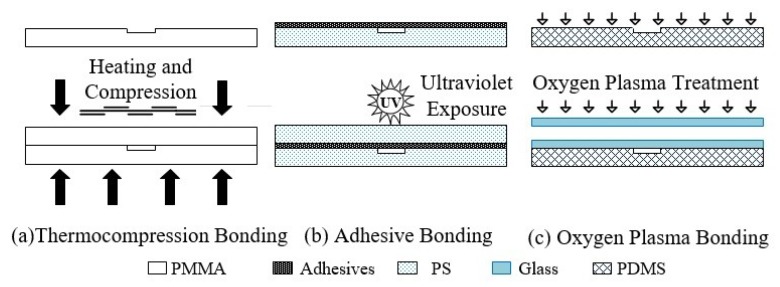
Common microfluidic chip bonding methods.

Except for paper-based microfluidic chips, which can use open flow channels, other types of microfluidic chips need to be covered with a layer of material (cover sheet) above the flow channel after the microstructure processing is completed to complete the flow channel closure, that is the bonding of the microfluidic chip. The cover sheet material and the substrate material can be the same material with the same thickness, and can also be used for bonding different types and thicknesses of materials for special purposes. Unlike the bonding between silicon and glass chips in ultra clean rooms using precision instruments, researchers have proposed various low-cost microfluidic chip bonding methods in recent years, mainly including thermal compression bonding, adhesive bonding, plasma surface treatment and laser welding, as shown in Figure 4.

### 4.6. Thermal Compression Bonding

Thermal compression bonding is an ideal bonding method for microfluidic chips based on PMMA, PC, PS, Glass, and other thermoplastic materials (Figure 4a). After the two layers of materials are contacted and aligned, chip bonding is completed by heating and pressurizing at the same time, where the heating temperature is slightly higher than the glass transition temperature (Tg) of thermoplastic plastics, and the pressure can be set according to the actual situation. Researchers have made a deep exploration in the field of microfluidic chip bonding by using the hot embossing method, and have completed the study of the bonding strength of PMMA/PMMA [66], PMMA/PS (Figure 4b) [67], Glass (Figure 4c) [37] materials at different temperatures and pressures. The failure of thermocompression bonding for thermoplastic materials is often caused by the collapse of microstructure during the bonding process, due to excessive temperature or pressure. In practical use, on the one hand, it is necessary to strictly control the setting of temperature and pressure, while on the other hand, it can also use oxygen plasma or ultraviolet light to pretreat the surface of polymer materials, to reduce the molecular weight of the surface to be bonded in order to reduce the glass transition temperature of the surface [68].

## 5. Application

Microfluidic chip technology has been widely used in the detection of pesticide residue, pathogenic bacteria, heavy metal and food additives (Table 1).

### 5.1. Detection of Pesticide Residue

Pesticides can effectively protect or improve the yield of crops. Although, during the last years, important consequences in health have been detected in people who consume foods that have been treated with pesticides. Some of these consequences are diarrhea, respiratory difficulties, alteration of the sleep. In fact, in China, the number of people who develop cancer by eating food containing pesticide residues is increasing by 15% every year [95]. At present, the rapid detection methods of pesticides can be roughly divided into the enzyme inhibition method, spectral detection method and chromatographic detection method, according to the principle [96,97,98]. The traditional instrumental analysis method not only has high sensitivity and selectivity, but also can detect a variety of pesticide residues at the same time, but this method has the problems of large equipment, high detection cost, low degree of automation, being time-consuming and requiring a large amount of reagents and consumables, which is difficult to meet the needs of the rapid on-site screening of a large number of samples [99]. Therefore, the portable, highly automated and low-cost pesticide residue detection equipment has become a research hotspot in recent years. Hossain et al. [69] prepared a paper chip for the detection of organophosphorus pesticide (OPS) residues in food and beverage by measuring the activity of acetylcholinesterase on filter paper using the inkjet printing technique.

Acetylcholinesterase can catalyze the hydrolysis of indophenol acetate into acetic acid and blue-purple indophenol, while OPS and carbamate pesticides can inhibit the activity of acetylcholinesterase, slow down the hydrolysis process of indophenyl acetate, where the color change is affected, and finally establishing the corresponding relationship between the color change of products and pesticide residues, realize the detection of pesticide residues. Liu et al. [70] developed a paper-based detector for the detection of dichlorvos based on the luminol–H_2_O_2_ chemiluminescence system, which has been successfully applied to the detection of trace dichlorvos in cucumber, tomato and cabbage, with the limit of detection (LOD) as 3.6 ng/mL, which is lower than that of gas chromatography.

Chemiluminescence (CL) has been proven to be an excellent tool for the detection of organophosphorite (OPS). The reaction of dichlorvos (DDV) with luminol and H_2_O_2_ can directly produce CL emissions [100]. Therefore, the luminol CL system can be used to detect DDV with high sensitivity. Liu Wei et al. [71] designed a novel molecularly-imprinted polymer (MIP) experimental device based on paper with CL for DDV detection. In this device, a certain amount of MIP was synthesized on the paper surface by the cutting method, and DDV was selectively printed on it. A mixture of luminol and hydrogen peroxide dripped onto the surface of the paper. Then DDV was oxidized by H_2_O_2_ to form unstable peroxophosphonate intermediate, which reacted with luminol to generate the CL signal, and the device had good selectivity for DDV. This method has been successfully applied to the determination of DDV in vegetables. In the linear range of 3.0 ng/mL–1.0 μg/mL, the LOD was 0.8 ng/mL. This device has the advantages of specificity and selectivity, high sensitivity, good selectivity and easy operation. The new experimental device is also suitable for on-site environmental detection of pesticides, drugs or environmental pollutants in remote areas. Wang et al. [72] developed a colorimetric micro device based on plug microfluidic technology for the detection of OPS. A new colorimetric technique is provided by using small volume stoppers and microfluidics, which reduces the consumption of expensive reagents, and improves the reproducibility and precision. The LOD of malathion, acephate, methamidophos and diazinone were 33 nM and 90 nM, respectively. 

In order to quickly detect pesticide residues in vegetable samples, Guo Yemin et al. [73] embedded the gold interdigital array microelectrode (IDAM) into PDMS microfluidic immunosensor chip. The microfluidic chip consists of a microchannel for detecting the inlet and outlet of the micro chamber. With chlorpyrifos as the model compound, the anti-chlorpyrifos monoclonal antibodies was immobilized on IDAM to produce sensitive impedance changes. The electrochemical impedance spectroscopy (EIS) was combined with the sensor to detect chlorpyrifos. Under the optimal conditions, the microfluidic immunosensor has the advantages of high sensitivity, low reagent consumption, miniaturization and automation. Meanwhile, the proposed microfluidic immunosensor can also be used for the direct analysis of real samples. Detection of pesticide residues has great significance in the production of vegetables and fruits. Trichlorfon is one of the OPS phosphates, which can cause harm to human health. Yang et al. [74] proposed a multilayer paper chip based on the principle of enzyme inhibition and internal heating. A heating layer was built in the paper-based chip to ensure that the reaction process with the enzyme was always at the optimal temperature. The device could directly judge whether the pesticide exceeds the standard according to the reaction color. Under the optimum conditions, the LOD of trichlorfon was 0.0406 mg/L, and it had good repeatability, specificity and stability. Compared with pesticide detector and gas chromatography-mass spectrometry, the detection method proposed in the study had high resolution and low cost. This new multi-layer, paper-based, microfluidic chip has potential in the simultaneous detection of pesticide residues in crop production. At present, the standard method for detection of trichlorfon is gas chromatography (GC). Compared with GC, the microfluidic, paper-based phosphorusdetection chip (μPPC) can provide direct and reliable results in a short time, and has higher portability and convenience. A novel μPPC was proposed for the rapid analysis of trichlorfon in OPS residues [75]. Combined with a semi-quantitative method, the LOD was 1.65 μg/mL, which provides a new method for the rapid detection of OPS.

### 5.2. Detection of Pathogenic Bacteria

Food pathogenic bacteria are the most important threat to food safety in the world. People who consume foods that have been treated with pathogenic bacteria can cause food poisoning. For this reason, it is so important to contain with methodologies that allow the detection and quantification of pathogenic bacteria in foods. In all foodborne diseases, the three most pathogenic bacteria are *Escherichia coli*, *Salmonella* and *Listeria monocytogenes*, and eating food contaminated by *E*. coli O157: H7 can cause hemorrhagic colitis and hemorrhagic uremic syndrome [101]. The conventional detection methods need to carry out the culture and enrichment of bacteria, which is time-consuming [102,103]. Various types of microfluidic systems have been developed to detect bacterial pathogens. However, most microfluidic systems need complex concentration steps. Jokerst et al. [76] developed a paper-based microfluidic chip technique for the detection of *E*. coli O157: H7, *L. monocytogenes* and *Salmonella* in ready-to-eat meat products, based on the color intensity change of the system after specific enzymes secreted by bacteria react with substrates, with the LOD of *E*. coli, *L. monocytogenes* and *Salmonella* being 106, 108 and 104 CFU/mL, respectively. The prepared paper-based microfluidic chip can detect the pathogenic bacteria with the concentration as low as 101 CFU/mL in ready-to-eat meat in 12 h or less, and the detection period was significantly smaller than that of the gold-labeled method, which reached the detection range of the standard method, and greatly shortens the detection time compared with the traditional medical method. A 3D microfluidic magnetic preconcentrator (3DμFMP), which does not need any components, has been developed for the detection of *E*. coli O157: H7 [77]. The device could detect *E*. *coli* O157: H7 in a large volume of sample solution with the ATP photometer, as low as 10 CFU of *E. coli* O157: H7 (colony forming unit)/mL. It could selectively accumulate EHEC O157: H7 at a concentration of 700 times in one hour. These results proved the feasibility of 3DμFMP as a preconcentrator to improve the detection limit of the existing bacterial detection system. Li Tianchan et al. [78] combined loop-mediated isothermal amplification (LAMP) based on microfluidic chip with a carbon nanotube (CNT) multilayer biosensor to detect *E*. *coli* O157: H7. After cultivation of *E. coli*, the captured bacteria could be released as needed by cutting the interaction between anti-*E. coli* O157: H7 antibody and bacteria. Antibody-functionalized carbon nanotube multilayers could capture, culture and release bacteria selectively, and then we analyzed the DNA concentration of the releasing bacteria with the LAMP microfluidic chip. The proposed sensing platform was capable of detecting concentrations as low as 1 CFU/mL, which is much more sensitive than the previously reported method. The sensing platform has the advantages of low cost and easy operation, and is a potential platform for the detection of *E*. coli O157: H7 in food safety.

In order to meet the needs of the rapid on-line or on-site detection of pathogens, Sun et al. [79] first described an eight-chamber lab-on-a-chip (LOC) system with integrated magnetic bead-based sample preparation and LAMP for the rapid and quantitative detection of *Salmonella* species in food. The system analyzed eight samples of eutrophic pork containing *Salmonella* in 40 min, and the LOD of each detection was as low as 50 cells. Kim et al. [80] used quantum dot nanoparticles to detect *Salmonella* cells. Super paramagnetic particles and microfluidic chips were used to separate and concentrate *Salmonella* in the samples. The LOD of the synthesized sensor in borate buffer and food extract was 10 (3) CFU/mL *Salmonella*. Fronczek et al. [81] developed a hand-held optical immunoassay device for the detection of *Salmonella* typhimurium in fresh poultry packaging. The Mie Scattering signal was generated in the microfluidic channel through the immunoagglutination reaction of *Salmonella* and carboxylated polystyrene particles, and then the combined anti *Salmonella* was read by a hand-held device. The LOD was 10 CFU/mL, demonstrating the suitability of this device for field assay.

### 5.3. Detection of Heavy Metal

Heavy metal elements in food can react strongly with protein and various enzymes in the human body, making them lose their activity, and when they accumulate to a certain concentration, they will cause acute or subacute poisoning, chronic poisoning and other hazards, and the excessive content of heavy metal elements in food occurs from time to time, which has a potential threat to the health of consumers, so it is necessary to detect the content of heavy metals in food [104]. Jayawardane et al. [82] have developed a disposable paper-based sensor for the determination of copper (II) in natural and waste water. Under weak acid conditions, the device can selectively identify Cu (II) from natural water and wastewater containing metal ions, such as Fe (III), Al (III), Zn (II), CD (II), Pb (II), Ca (II), Mg (II) and Ni (II). The detection limit and quantification limit of Cu (II) are 0.06 and 0.21 mg/L respectively, and the linear range is 0.1–30 mg/L, which is comparable to the detection limit of atomic absorption spectrophotometry. The method has been successfully applied to the determination of Cu (II) in tap water and tailing water. Shi et al. [83] developed an electrochemical paper-based microfluidic chip based on square wave anodic stripping voltammetry for the simultaneous determination of Pb (II) and Cd (II) in carbonated beverages. The LOD of Pb and Cd in carbonated beverages were 2 μg/L and 2.3 μg/L, respectively, which were comparable to those of graphite furnace atomic absorption spectrophotometry. Zhang et al. [84] combined with a fluorescently labeled, single-stranded DNA (ssDNA) functionalized graphene oxide sensor to develop a low-cost, simple, paper-based microfluidic device that can be used to simultaneously determine multiple chemical contaminants in food. The biosensor has been successfully applied to the simultaneous determination of heavy metals (such as mercury and silver) and aminoglycoside antibiotics in foods. The recoveries of mercury silver and aminoglycoside antibiotics were 87%–116%, 91%–126% and 95%–101% respectively. The LOD of mercury, silver and aminoglycoside antibiotics were 121, 47 and 153 nmol/L. 

A new paper-based copper ion colorimetric sensor with high selectivity and sensitivity was developed to measure the catalytic etching of silver nanoplates (agnpls) based on thiosulfate [85]. Cu^2+^ was detected in the range of 0.5–200 ng/mL (R^2^ = 0.9974), and LOD was 0.3 ng/mL. This method has been successfully applied to the determination of Cu^2+^ in water, food, blood and other practical samples. In order to detect lead (Pb^2+^) rapidly and sensitively, Fan Chunhui et al. [86] designed a portable and power-free microfluidic device, and used 11-mercaptoundecanoic acid (MUA) modified gold nanoparticles (AuNPs) as detection probes. MUA-AuNPs aggregated in the presence of Pb^2+^ to form a chelation mechanism, and it could be clearly seen that the color of the solution changes from red to purple, obviously due to the influence of plasma coupling. The aggregates deposited on the surface of PDMS microfluidic chips and form dark lines along the laminar flow in the zigzag microchannels. This visual effect could be observed with the naked eye through a microscope or a drop of water as a magnifier. The LOD was 10 μM Pb^2+^, which was competitive with the traditional physicochemical quantitative methods. Santangelo et al. [87] demonstrated a new sensing platform based on an epitaxial graphene sensor coupled to a 3D printing microfluidic chip for real-time detection of heavy metals. Due to the extreme sensitivity of the material, low trace Pb^2+^ could be detected by using the EG sensor and 3D printing microfluidic chip. The LOD was 95 nM, far lower than the World Health Organization (WHO) recommended limit for lead content in drinking water. Qi Ji et al. [88] proposed a novel, three-dimensional (3D) origami ion imprinted polymers microfluidic paper-based chip device for specific, sensitive and multiplex detection of Cu^2+^ and Hg^2+^ ions by combining microfluidic and ion imprinting technology. In this device, CdTe quantum dots (QDs) were grafted onto the surface of glass fiber paper, and the photoluminescence energy of QDs would be transferred to their ion imprinted QD complexes and caused fluorescence changes. The designed 3D origami microfluidic paper-based analyzer (μPADs) had a “Y” type channel, which could be used for the selective identification of Cu^2+^ and Hg^2+^ samples, and had good selectivity and sensitivity. The results showed that the sensor has a good linear relationship in the range of 0.11–58.0 μg/L, the LOD was 0.035 μg/L, the linear range of mercury is 0.26–34.0 μg/L, and the LOD was 0.056 μg/L. The device has been successfully used in the simultaneous detection of actual water samples. 

### 5.4. Detection of Food Additives

Adding pigments to food has become a routine practice to enhance or change the color of food, making it more attractive to consumers. However, most of these pigments may cause potential harm to human health, even teratogenic and carcinogenic [105]. Therefore, it is necessary to establish a detection and analysis method for pigments in food. A novel, paper-based microfluidic chip for the separation and detection of pigments was developed [89]. The pigments in the compound beverage were separated, concentrated and detected by surface-enhanced Raman spectroscopy (SERS) with the functionalized paper-based carriers, after the polyelectrolytes poly (allylamine hydrochloride) and poly (sodium styrene sulfonate) were modified onto silver nanoparticle-based filter paper through a simple procedure. The separation and concentration of the analytes were achieved by surface chemical gradients and electrostatic interaction generated by the polyelectrolyte-coated paper. The LOD of sunset yellow and tartrazine in grape juice and orange juice were 10^−5^ mol/L and 10^−4^ mol/L, respectively. Nitrite, as a kind of food additive, is often used in the production of meat products to increase the freshness of meat, inhibit microorganisms, and help to maintain the structure and nutritional value of meat products, but excessive intake may cause poisoning or death [106,107]. Jayawardane et al. [90] developed a disposable microfluidic paper-based analytical device based on inkjet printing for the determination of nitrite and nitrate. Under the optimum conditions, the LOD of nitrite was 1.0 μm, the LOD of nitrate was 19 μm. The device has the advantages of user convenience, friendly environment, and being suitable for field measurement. Fujii et al. [91] used a new microchip fluorescence detection device to study the method for the determination of sulfite and nitrite with N-(9-acridinyl) and 2,3-diaminonaphthalene (DNA). The sulfite and nitrite in environmental samples were simultaneously determined by using a polymer microchip analysis system. The calibration curves of sulfite and nitrite showed a linear relationship. The relative standard deviation (RSD) of the four determinations were 2.1% (20 microM sulfite) and 1.3% (20 microM nitrite), respectively. This method could be used to recover sulfite and nitrite from environmental samples. Cardoso et al. [92] reported μPADs combined with colorimetry for the determination of nitrite in clinical, food and environmental samples. Eight circular detection areas and one central area of μPADs were prepared by stamping in the geometry container, and the sample inlet was connected by microfluidic channel. The device has successfully determined the concentration of nitrite in ham, sausage and river water. The LOD was 5.6 μM. There was no significant difference between the concentration measured by μPADs and that measured by spectrophotometry.

Liu et al. [93] proposed an integrated microfluidic platform based on the Janovsky reaction theory, which consists of a microfluidic paper-based analysis device (PAD) and a portable benzoic acid concentration detection system. The 3,5-dinitrobenzoic acid obtained by the reaction of benzoic acid sample with KNO_3_ and H_2_SO_4_ was dropped in the reaction zone of the chip. Then PAD was transferred to a portable detection system and heated to induce the Janovsky reaction. The color change of the detection area could be observed by a Complementary Metal Oxide Semiconductor (CMOS) camera. The reaction color image was transmitted to the smartphone through the connector, and the concentration of the benzoic acid sample was evaluated by analyzing the RGB color intensity of the image using a self-made application program. The platform has been used to detect benzoic acid in 21 kinds of commercial food samples. The results showed that the deviation between the measured value of the concentration and that of the standard high-performance liquid chromatography was less than 6.6%. In the food and beverage industry, food additives such as glucose and fructose are widely used to improve or enhance the flavor or color of food or beverage. Although most of the food additives are non-toxic, a large number of food additives may cause health problems, and some of them may produce toxicity to a certain extent. Lawrence et al. [94] established a method of current detection of glucose based on screen-printed paper-based microfluidic chip. They developed an amperometric glucose biosensor by immobilizing glucose oxidase on a “green” biocompatible fiber-based paper disk on a screen-printed carbon electrode. The prepared paper disk has good hydrophilicity, which provides a biocompatible microenvironment for maintaining the catalytic activity of glucose oxidase. 

The method utilized ferrocenyl carboxylic acid as a modulator for the catalytic oxidation of glucose. The biosensor has been successfully applied to the selective determination of glucose in different commercial carbonated drinks with a detection limit of 0.18 mmol/L. The results obtained were consistent with the HPLC method.

## 6. Conclusions

As a new technology, the microfluidic chip can concentrate multiple steps of sample detection on a small chip, integrate these operations through the size and curvature of the flow channel, micro-valves, cavity design, and ultimately make the whole detection integration miniaturization and automation. Because microfluidics can be designed to be multi-channel, the samples to be detected can be shunted to multiple reaction units at the same time through the microfluidic network, which has the characteristics of high throughput compared with conventional item-by-item detection. In addition, microfluidic technology also has the advantages of the low consumption of detection reagents and sample.

Microfluidic chip technology also have some limitations. For microfluidic immunoassay chips, the problem is that the analysis chips are disposable, which cannot give full play to the advantages of the microfluidic analysis platform that can be used for many times, leading to high detection costs. In addition, microfluidic chips require high technology, such as antibody immobilization, which is also a key issue. The integration of microfluidic chips and peripheral devices, such as automatic analysis and display devices, is also a difficult problem to be solved.

Aptamer is a new type of molecular recognition probe in the field of food detection, which has high affinity and binding specificity to target molecules. However, the literature on the development of an aptamer-based microfluidic platform is very limited, because it is a recently developed field. In the future, microfluidic analysis system combined with nanomaterials and new biomolecules will be able to provide a fast, powerful and sensitive food analysis and detection device, which will become a new development trend [108]. Paper-based microfluidic chip technology has made a series of important progress in the detection of food ingredients, pesticide residues, pathogenic bacteria, heavy metals, food additives and other aspects, with its advantages of simple production, low cost, portable, easy storage and transportation, simple and fast operation, and has shown great application prospects in the field of food safety detection [109]. The industrialization of microfluidic chip will become the development trend in the future. In the next few years, microfluidic chips will enter a more in-depth basic research, widely expand the application field, and the depth industrialization of the transition period.

## Figures and Tables

**Table 1:** Summary of microfluidic chip technology in food safety sensing.

Target	Chip Materials	Detection Method	LOD	Real Sample Application	Reference
OPS	paper	colorimetric	-	food, beverage	[69]
DDV	paper	CL	3.6 ng/mL	cucumber, tomato and cabbage	[70]
DDV	paper	CL	0.8 ng/mL	vegetables	[71]
OPS	-	colorimetric	33 nM, 90 nM	-	[72]
chlorpyrifos	PDMS	EIS	-	vegetable real samples	[73]
trichlorfon	paper	colorimetric	0.0406 mg/L	-	[74]
OPS	paper	semi quantitative	1.65 μg/mL	-	[75]
*E*. coli O157: H7, *L. monocytogenes* and *Salmonella*	paper	colorimetric	10^6^, 10^8^ and 10^4^ CFU/mL	ready-to-eat meat	[76]
*E*. coli O157: H7	plastic	spectrophotometry	10 CFU/mL	blood	[77]
*E*. coli O157: H7	glass	LAMP	1 CFU/mL	-	[78]
*Salmonella*	-	LAMP	50 cells	pork	[79]
*Salmonella*	silicon	electrochemical	10^3^ CFU/ml	borate buffer and food extract	[80]
*Salmonella*	PDMS	optical immunoassay	10 CFU/ ml	fresh poultry packaging	[81]
Cu (II)	paper	-	0.06 mg/L	tap water and tailing water.	[82]
Pb (II), Cd (II)	paper	electrochemical	2 μg/L, 2.3 μg/L	carbonated beverages	[83]
Hg(II), Ag(I)	paper	fluorescence	121 nM, 47 nM	-	[84]
Cu(II)	paper	Colorimetric	0.3 ng/mL	Drinking water, ground water, tomato, rice	[85]
Pb(II)	PDMS	electrochemical	10 μM	-	[86]
Pb(II)	-	electrochemical	95 nM	-	[87]
Cu(II), Hg(II)	paper	fluorescence	0.035 μg/L, 0.056 μg/L	water samples	[88]
sunset yellow, tartrazine	paper	surface-enhanced Raman spectroscopy	10^−5^ M, 10^−4^ M	Grape juice, orange juice	[89]
nitrite, nitrate	paper	colorimetric	1.0 μM, 19 μM	Tap water, mineral water, pond water	[90]
Sulfite, nitrite	polymer	fluorescence	-	-	[91]
nitrite	paper	colorimetric	5.6 μM	Ham, sausage, preservative water	[92]
benzoic acid	paper	-	-	commercial food samples	[93]
glucose	paper	electrochemical	0.18 mM	commercial carbonated drinks	[94]
